# The effect of and correction for through‐slice dephasing on 2D gradient‐echo double angle $B_{1}^{+}$ mapping

**DOI:** 10.1002/mrm.29966

**Published:** 2023-12-29

**Authors:** Gabriela Belsley, Damian J. Tyler, Matthew D. Robson, Elizabeth M. Tunnicliffe

**Affiliations:** ^1^ Oxford Centre for Clinical Magnetic Resonance Research, Radcliffe Department of Medicine University of Oxford Oxford UK; ^2^ Perspectum Oxford UK

**Keywords:** $B_{1}^{+}$ mapping, slice profile effects, $T_{1}$ mapping, through‐slice dephasing

## Abstract

**Purpose:**

To show that $B_{0}$ variations through slice and slice profile effects are two major confounders affecting 2D dual angle $B_{1}^{+}$ maps using gradient‐echo signals and thus need to be corrected to obtain accurate $B_{1}^{+}$ maps.

**Methods:**

The 2D gradient‐echo transverse complex signal was Bloch‐simulated and integrated across the slice dimension including nonlinear variations in $B_{0}$ inhomogeneities through slice. A nonlinear least squares fit was used to find the $B_{1}^{+}$ factor corresponding to the best match between the two gradient‐echo signals experimental ratio and the Bloch‐simulated ratio. The correction was validated in phantom and in vivo at 3T.

**Results:**

For our RF excitation pulse, the error in the $B_{1}^{+}$ factor scales by approximately 3.8% for every 10 Hz/cm variation in $B_{0}$ along the slice direction. Higher accuracy phantom $B_{1}^{+}$ maps were obtained after applying the proposed correction; the root mean square $B_{1}^{+}$ error relative to the gold standard $B_{1}^{+}$ decreased from 6.4% to 2.6%. In vivo whole‐liver $T_{1}$ maps using the corrected $B_{1}^{+}$ map registered a significant decrease in $T_{1}$ gradient through slice.

**Conclusion:**

$B_{0}$ inhomogeneities varying through slice were seen to have an impact on the accuracy of 2D double angle $B_{1}^{+}$ maps using gradient‐echo sequences. Consideration of this confounder is crucial for research relying on accurate knowledge of the true excitation flip angles, as is the case of $T_{1}$ mapping using a spoiled gradient recalled echo sequence.

## 1 INTRODUCTION

Several applications rely on the accurate knowledge of the true flip angles (FAs) exciting the spins,^1^, ^2^, ^3^, ^4^, ^5^, ^6^ including $T_{1}$ mapping using the variable flip angle (VFA) spoiled gradient recalled echo (SPGR) sequence. The double‐angle method (DAM) for $B_{1}^{+}$ mapping^7^ can be implemented using widely available and scanner‐agnostic pulse sequences such as gradient recalled echo (GRE) with an echo‐planar imaging (EPI) readout.

The impact of through‐slice dephasing on multi‐echo GRE data and $R_{2}^{*}$ measurements is well recognized in the literature.^8^, ^9^, ^10^, ^11^ It has been assumed that $B_{0}$ inhomogeneity should not be relevant to GRE‐based DAM $B_{1}^{+}$ maps^7^ because the two signals, acquired at excitation FAs of 2α and α, have the same echo time (TE). However, the large FAs typically required in the DAM^1^, ^12^ mean that through‐slice $B_{0}$ variation can affect the measured ratio.

On‐resonance in the small angle approximation, the phase roll accrued due to the slice select gradient is set to zero by the rephasing gradient. However, for FAs above 30°, where the small angle approximation breaks down, even in the absence of $B_{0}$ field inhomogeneities, the phase accrued during the slice‐select gradient is not completely refocused by the refocusing gradient for the usual convention of using a refocusing gradient with half the moment of the slice select gradient.^13^ This results in a residual phase roll across the slice. The degree of signal loss that results depends on the shape of the slice profile, being greater for slice profiles with more energy farther from the slice center. Consequently, the ratio of the integrated complex signal in the DAM $B_{1}^{+}$ factor calculation will be altered compared to the case of equal phase roll for the two FAs.

Using a 2D multi‐slice implementation of the GRE‐EPI for DAM $B_{1}^{+}$ mapping suffers from slice profile effects.^12^, ^14^ For a constant $B_{0}$ offset, integrating the Bloch‐simulated complex signal through slice,^15^ before taking the ratio of the signals in the DAM, will account for the phase roll differences between 2α and α as well as the different slice profiles.

However, $B_{0}$ inhomogeneities along the slice direction impose an additional dephasing of the spins on top of the dephasing from the slice‐select gradient. Organs located at interfaces with large susceptibility differences suffer from large $B_{0}$ inhomogeneities, reaching values at the liver dome of 200 Hz at 3T. These inhomogeneities in the liver decrease with distance from the lung, creating a $B_{0}$ gradient through slice ($\nabla_{z}B_{0}$). We hypothesized that the existence of this $\nabla_{z}B_{0}$ would alter the phase roll profile for each FA and lead to a dependence of the calculated $B_{1}^{+}$ factor on the $\nabla_{z}B_{0}$.

In this work, the effect of a $\nabla_{z}B_{0}$ on the $B_{1}^{+}$ factor was studied. Simulations using linear $\nabla_{z}B_{0}$ were used to estimate the error introduced in the $B_{1}^{+}$ map when neglecting this effect. A novel correction for the $\nabla_{z}B_{0}$ effects on the GRE‐EPI $B_{1}^{+}$ map is proposed, resulting in accurate $B_{1}^{+}$ maps using widely available pulse sequences. Validation of the proposed corrections to the $B_{1}^{+}$ map was conducted in a phantom. The correction method was also applied to the nominal FAs in VFA SPGR $T_{1}$ maps of the liver in vivo.

## 2 METHODS

### 2.1 Simulations

Bloch equations with 201 points covering the slice profile were used to simulate^16^ transverse signals at FAs of 130° and 65°^12^ using the vendor's Hamming‐windowed sinc GRE‐EPI excitation pulse (FWHM of 0.55 ms for a 3.2 ms long simulation window). Signals were simulated on‐resonance, with a constant off‐resonance and with a $\nabla_{z}B_{0}$ varying between −45 Hz/cm and 45 Hz/cm, in steps of 5 Hz/cm. Note that these $\nabla_{z}B_{0}$ are small relative to the slice‐select gradient (2.54 kHz/cm); thus, there is no detectable slice distortion. The code is available here: https://github.com/gabrielaBelsley/ThroughSliceDephasing_2DGRE (SHA‐1 hash c694 101).

The effect of the $\nabla_{z}B_{0}$ on the $B_{1}^{+}$ factor was quantified through the deviation of the estimated $B_{1}^{+}$ factor from true $B_{1}^{+}$ biases of 0.59, 1, and 1.14. These correspond to the range of liver $B_{1}^{+}$ factors observed in vivo at 3T.^17^

### 2.2 Image acquisition

Imaging data were acquired on a phantom and 10 healthy volunteers, five male and five female, on a 3T Prisma (Siemens Healthineers, Germany) scanner. Volunteers were scanned according to our institution's ethical practices and gave informed consent.

A 2D multi‐slice GRE single‐shot EPI was used for the $B_{1}^{+}$ mapping with fat saturation and nominal FAs of $65^{\circ }$ and $130^{\circ }$.^18^ Acquisition parameters were FOV = 450×366 $mm^{2}$, matrix = 64×52, 15 slices interleaved, slice thickness/spacing 8/2 mm, TE/TR = 11/10000 ms, linear phase encoding, no acceleration or phase partial Fourier, bandwidth (BW) 3906 Hertz/pixel, and acquisition time 10 s breath‐hold.

A 2D multi‐slice double‐echo spoiled GRE acquisition was acquired to compute a $B_{0}$ map. The $B_{0}$ map was used for distortion correction of the GRE‐EPI images through *fsl fugue* ^19^, ^20^ and modeling of $\nabla_{z}B_{0}$ in the $B_{1}^{+}$ map calculation. Acquisition parameters were TR/TE1/TE2 = 20/4.78/7.17 ms, FA=$\;15^{\circ }$, FOV = $450\times 380\;mm^{2}$, matrix = 64×54, slice thickness/spacing 8/2 mm, 15 slices, monopolar readout gradients, BW = 630 Hz/pixel, GRAPPA^21^ two times acceleration, and acquisition time 8.6 s breath‐hold.

The $T_{1}$ contrast of the liver tissue was obtained through a 3D VFA SPGR with Dixon^22^ fat/water separation. Acquisition parameters were FOV = $450\times 366\times 144\;mm^{3}$, matrix = 320×260×48, TR/TEs = 4.1/(1.23,2.46) ms, and BW = 1040 Hz/pixel. Data were acquired at nominal FAs of $2^{\circ }$, $2^{\circ }$, $15^{\circ },$ and $15^{\circ }$,^18^ each during a 15 s breath‐hold. Caipirinha^23^ with 3× acceleration along the slice direction with 24 separate GRE reference lines was used. Spatial saturation was turned off because it perturbs the steady‐state signal.

Gold standard (GS) $B_{1}^{+}$ and $T_{1}$ maps were performed on the phantom. The GS $B_{1}^{+}$ map consisted of a 3D nonselective DAM GRE acquisition with a long TR of 10 s for full relaxation of longitudinal magnetization and without an EPI readout. For the GS $T_{1}$ map, a slice‐selective inversion recovery (IR) spin echo (SE) was used with inversion times logarithmically increasing between 25 ms and 5000 ms. Acquisition details, maps, and linear fits between the SPGR $T_{1}$ and GS $T_{1}$ are in the Supporting Information.

### 2.3 DAM $B_{1}^{+}$ map corrected for slice profile effects

The DAM $B_{1}^{+}$ mapping^7^ takes the ratio between two signals from fully relaxed spins, $S_{2}$ and $S_{1}$, acquired respectively at nominal FAs 2α and α to estimate the true FA exciting the spins:

(1) $$\alpha \left(r\right)=\arccos\left(\left|\frac{S_{2}\left(r\right)}{2S_{1}\left(r\right)}\right|\right),$$

where r is the voxel slice center coordinate position.

A nonuniform slice profile invalidates Equation 1. To correct for slice profile effects, the Bloch‐simulated complex transverse signal was integrated across the slice for each FA from 1° to 360°. The ratio was taken between the absolute value of the integrated complex signals at 2α and α (Equation 2) to generate a $B_{1}^{+}$ lookup table (LUT) of the ratio (*R*) as a function of the FA (Figure S1).

(2) $$R\left(r\right)=\frac{S_{2}\left(r\right)}{S_{1}\left(r\right)}=\frac{\left|\int \mathrm{dz}{\left(\left|M_{\mathrm{xy}}\left(z\right)\right|e^{i\phi_{M_{\mathrm{xy}}}\left(z\right)}\right)}_{2\alpha }\right|}{\left|\int \mathrm{dz}{\left(\left|M_{\mathrm{xy}}\left(z\right)\right|e^{i\phi_{M_{\mathrm{xy}}}\left(z\right)}\right)}_{\alpha }\right|}.$$

At each pixel, the ratio of the signal acquired at FAs 2α and α was linearly interpolated from the LUT to obtain the true FA exciting the spins. The $B_{1}^{+}$ correction factor is the ratio between the true FA and the nominal FA prescribed at the scanner.

### 2.4 $B_{0}$ gradient through slice correction to the slice profile‐corrected $B_{1}^{+}$ map

The correction for the $\nabla_{z}B_{0}$ effect was based on Bloch simulations^16^ of the signal, using the RF excitation pulse, slice‐select gradient ($G_{\mathrm{SS}}$), and slice refocusing gradient ($G_{\mathrm{Ref}}$) information, and including varying off‐resonances in the slice direction. To determine the through‐slice $B_{0}$ at a 1 mm spatial resolution for each pixel in the $B_{1}^{+}$ map, the $B_{0}$ values across the liver along the slice direction were fit to a cubic spline.^24^ The Bloch‐simulated complex signals, for FAs 2α and α, were calculated at a 1 mm spatial resolution over a spatial extension of ±1cm from slice center for each voxel. The transverse signal immediately after the RF excitation pulse, calculated with the interpolated off‐resonance, was propagated until TE, including free precession at the corresponding off‐resonance and $T_{1}/T_{2}$ relaxation with values of 900/30 ms, respectively. Lastly, the complex signal at TE for each voxel was integrated across the slice dimension, and the ratio was taken between the absolute value of the two signals (Equation 2).

A nonlinear least squares (NLLS) fit was used to find the $B_{1}^{+}$ factor corresponding to the Bloch‐simulated ratio ($\text{Rati}o_{\mathrm{Sim}}$) that best matched the ratio between the distortion‐corrected GRE‐EPI images acquired at nominal FAs of 130° and 65° ($\text{Rati}o_{\mathrm{acq}}$) for each pixel (Equation 3).

(3) $$\arg\min_{\left\{B_{1}^{+}\right\}}{\left(\text{Rati}o_{\mathrm{acq}}-\text{Rati}o_{\mathrm{Sim}}\left(\nabla_{z}B_{0}\left(z\right),G_{\mathrm{SS}},G_{\mathrm{Ref}},\mathrm{RF},\mathrm{TE}\right)\right)}^{2}.$$

To speed up the NLLS fit convergence, the starting point for the $B_{1}^{+}$ correction factor was the $B_{1}^{+}$ map factor corrected only for slice profile effects, using the LUT described in section C. Moreover, the $B_{1}^{+}$ was only calculated for voxels within the liver by applying a liver mask. The mask was manually delineated for each slice on the high spatial‐resolution SPGR FA $15^{{}^{\circ}}$ and down‐sampled to the $B_{1}^{+}$ and $B_{0}$ resolutions.

### 2.5 $T_{1}$ Mapping calculation and analysis

The corrected $B_{1}^{+}$ map was interpolated to the SPGR spatial resolution and multiplied by the nominal SPGR FAs to obtain the true excitation FAs. A correction for incomplete spoiling was applied to the SPGR signal, through extended phase graphs simulations,^25^ which was then fit to the steady‐state SPGR function through a NLLS regression.^26^

$T_{1}$ maps were analyzed by defining three 8‐pixel diameter circular regions of interest (ROIs) per slice. ROIs were placed in vessel and bile‐free areas, avoiding the liver edges. A linear fit between the mean $T_{1}$ values, extracted from each ROI, and the slice dimension before and after the $\nabla_{z}B_{0}$ correction was used to quantify the effect of the $B_{0}$ gradient on liver $T_{1}$. The resulting slope multiplied by the number of slices gives the $T_{1}$ variation ($∆T_{1}$) in ms across the liver.

A linear mixed model was used to assess the $\nabla_{z}B_{0}$ effect on the $T_{1}$ values before and after the $\nabla_{z}B_{0}$ correction. The linear mixed model was applied at the slice level—that is, each mean $T_{1}$ across the slice is one data point—over all the 10 volunteers. In Equation 4, $\beta_{0}$ and $\beta_{1}$ are the regressor coefficients for the intercept and the $\nabla_{z}B_{0}$, respectively; *(1|volunteer)* represents the random effects term in the intercept and ε is the error term.

(4) $$T_{1}=\beta_{0}+\beta_{1}\frac{\partial B_{0}}{\partial z}+\left(1|\text{volunteer}\right)+\varepsilon .$$

## 3 RESULTS

### 3.1 Simulations: On‐resonance and constant off‐resonance phase roll at M_xy_ signals α and 2α

**FIGURE 1:**
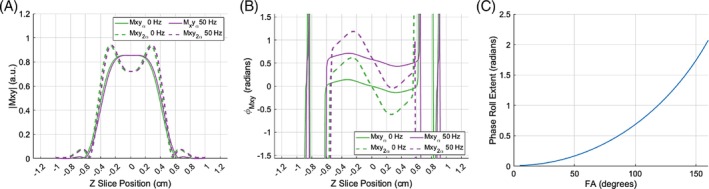
(A) Absolute value of the transverse magnetization in arbitrary units (a.u.) for RF excitation FAs of α equal to 65° (solid line) and 2α equal to 130° (dashed line) on‐resonance (green) and with a constant off‐resonance of 50 Hz (purple). (B) Phase of the transverse magnetization on‐resonance for 2α (dashed green line) and α (solid green line). A constant $B_{0}$ off‐resonance shifts the phase by a constant offset (purple lines). The signals correspond to a time moment immediately after the rephasing gradient. (C) Phase roll extent quantified as the difference between the maximum and minimum phase accrual across slice positions varying between −0.5 cm and 0.5 cm for FAs varying between 5° and 160° on‐resonance. FA, flip angle.

Figure 1B shows the residual phase on‐resonance post‐excitation pulse, which is larger for 2α. A constant off‐resonance for all the z positions introduces an overall constant phase offset (purple lines), which is equal for both FAs and will not affect the $B_{1}^{+}$ ratio. Figure 1C shows the breakdown in the small angle approximation, confirming that phase roll increases with FA when the rephasing gradient moment is half the slice‐select gradient moment.^13^

### 3.2 Simulations: Effect of a $\nabla_{z}B_{0}$ on the 2D GRE‐EPI DAM $B_{1}^{+}$

**FIGURE 2:**
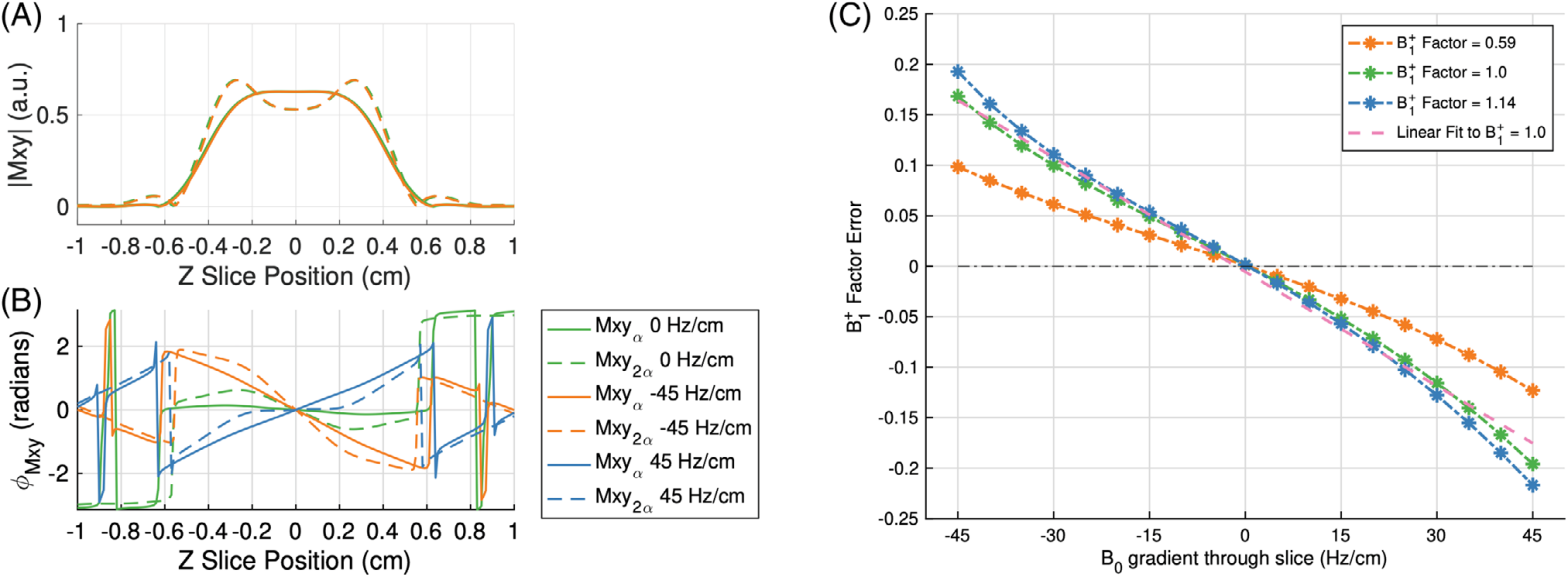
(A) Absolute transverse signal for α equal to 65° (solid line) and 2α (dashed line) on‐resonance (green) and for a $B_{0}$ gradient in the slice direction of −45 Hz/cm (orange) and +45 Hz/cm (blue). (B) Phase in radians for the transverse signal on‐resonance (green) at TE, with a negative $B_{0}$ gradient of −45 Hz/cm (orange) and a positive $B_{0}$ gradient of +45 Hz/cm (blue). The phase accrual for 2α (dashed green line) is larger than for α (solid green line) on‐resonance. A negative $B_{0}$ gradient across the slice position reinforces the dephasing of the negative slice‐select gradient, whereas a positive $B_{0}$ gradient results in a phase accrual in the opposite direction. (C) $B_{1}^{+}$ factor error ($\left|B_{1,\mathrm{slice}\mathrm{profile}}^{+}-B_{1,\mathrm{true}}^{+}\right|$) as a function of $B_{0}$ gradient through slice for three different $B_{1}^{+}$ factors of 0.59 (orange), 1.0 (green), and 1.14 (blue). The error increases for larger absolute $B_{0}$ gradients as well as increasing $B_{1}^{+}$ factors. A linear fit (pink dashed line) to the blue curve shows that at $B_{1}^{+}$=1, the error in $B_{1}^{+}$ factor scales by approximately 3.8% per every 10 Hz/cm variation in $B_{0}$ along the slice direction.

On‐resonance, the phase roll for 2α is larger than α (dashed vs. solid green line in Figure 2B). In the presence of a negative off‐resonance gradient (orange lines in Figure 2B), the preexisting phase roll is reinforced. The phase difference for the two peaks of the absolute transverse magnetization at 2α is 0.92π, close to the condition for complete destructive interference. This destructive interference reduces the overall integrated signal for the 2α pulse. In contrast, for the α pulse the largest contribution comes from a region around the slice center where the phase roll remains small. Consequently, the ratio is less than that on‐resonance. When the off‐resonance gradient is of opposite sign to the slice‐select gradient, the phase roll for the 2α pulse is smaller near the slice center compared to on‐resonance (green dashed line vs. blue dashed line in Figure 2B), resulting in a larger integrated signal for 2α. The overall effect of a positive off‐resonance gradient is an increased ratio compared to on‐resonance. Failure to address variations in the ratio due to the $\nabla_{z}B_{0}$ will result in over‐ or underestimation of the $B_{1}^{+}$ factor. The $B_{1}^{+}$ factor error scales by approximately 3.8% per every 10 Hz/cm variation in $B_{0}$ along the slice direction, at a $B_{1}^{+}$ factor of 1.

### 3.3 Phantom

**FIGURE 3:**
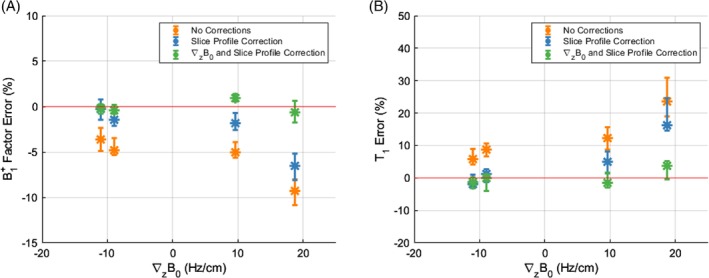
(A) $B_{1}^{+}$ factor error in the phantom $B_{1}^{+}$ map, normalized by the $B_{1}^{+}$ factor gold standard value, without any corrections (orange), with slice profile correction (blue), and with the $B_{0}$ gradient through slice correction added to the slice profile correction (green) for four GRE‐EPI slices. Without any corrections, the median $B_{1}^{+}$ factor error was −5.2% with an IQR of (−7.4 −3.7)% over the four slices. Using only slice profile correction, the median $B_{1}^{+}$ factor error was −1.8% with an IQR of (−4.5 −0.5)% over the four slices. Including the correction for off‐resonance variations through slice, the $B_{1}^{+}$ factor error decreased to 0.01% (−0.8 0.8)% over the four slices. (B) Percentage $T_{1}$ error, normalized by the IR SE, in the phantom $T_{1}$ map for four VFA‐SPGR slices matching the GRE‐EPI slices in (A). The colors are the same as described in (A). Without any corrections, the median and IQR $T_{1}$ error was 10.5% (6.7 17.5)% over the four slices. Using only slice profile correction, the median and IQR $T_{1}$ error was 2.6% (−0.4 10.2)% over the four slices. Including the correction for off‐resonance variations through slice, the $T_{1}$ error decreased to −0.3% (−2.5 2.1)% over the four slices. GRE, gradient recalled echo; EPI, echo‐planar imaging; IQR, interquartile range; IR, inversion recovery; SE, spin echo; SPGR, spoiled gradient recalled echo; VFA, variable flip angle.

Figure 3A shows the $B_{1}^{+}$ factor error for the phantom, measured against a $B_{1}^{+}$ GS 3D DAM, before (blue) and after (green), applying the $\nabla_{z}B_{0}$ correction for four different slices. The median error decreased from −1.8% to 0.01%, and the root mean square error relative to the GS $B_{1}^{+}$ decreased from 6.4% to 2.6%. The interquartile range using only slice profile correction was (−4.5 −0.5)%, becoming approximately three times tighter, (−0.8 0.8)% after including corrections for $\nabla_{z}B_{0}$.

Figure 3B shows the error in $T_{1}$ values for the phantom, measured against the GS $T_{1}$, before and after applying the $\nabla_{z}B_{0}$ corrections. The median $T_{1}$ error decreased from 2.6% to −0.3%. The interquartile range using only slice profile correction was (−0.5 10.2)% and decreased by more than a half, (−2.5 2.1)%, after including corrections for $\nabla_{z}B_{0}$. The slice with the largest median $B_{1}^{+}$ factor error of −6.6% (giving a median $T_{1}$ error of 16.3%) before correction had the largest $\nabla_{z}B_{0}$ of 18.7 Hz/cm. After applying the $\nabla_{z}B_{0}$ correction, this slice had the largest $T_{1}$ error reduction. Phantom $B_{1}^{+}$ and $T_{1}$ maps are shown in the Supporting Information (Figure S6).

### 3.4 In vivo

**FIGURE 4:**
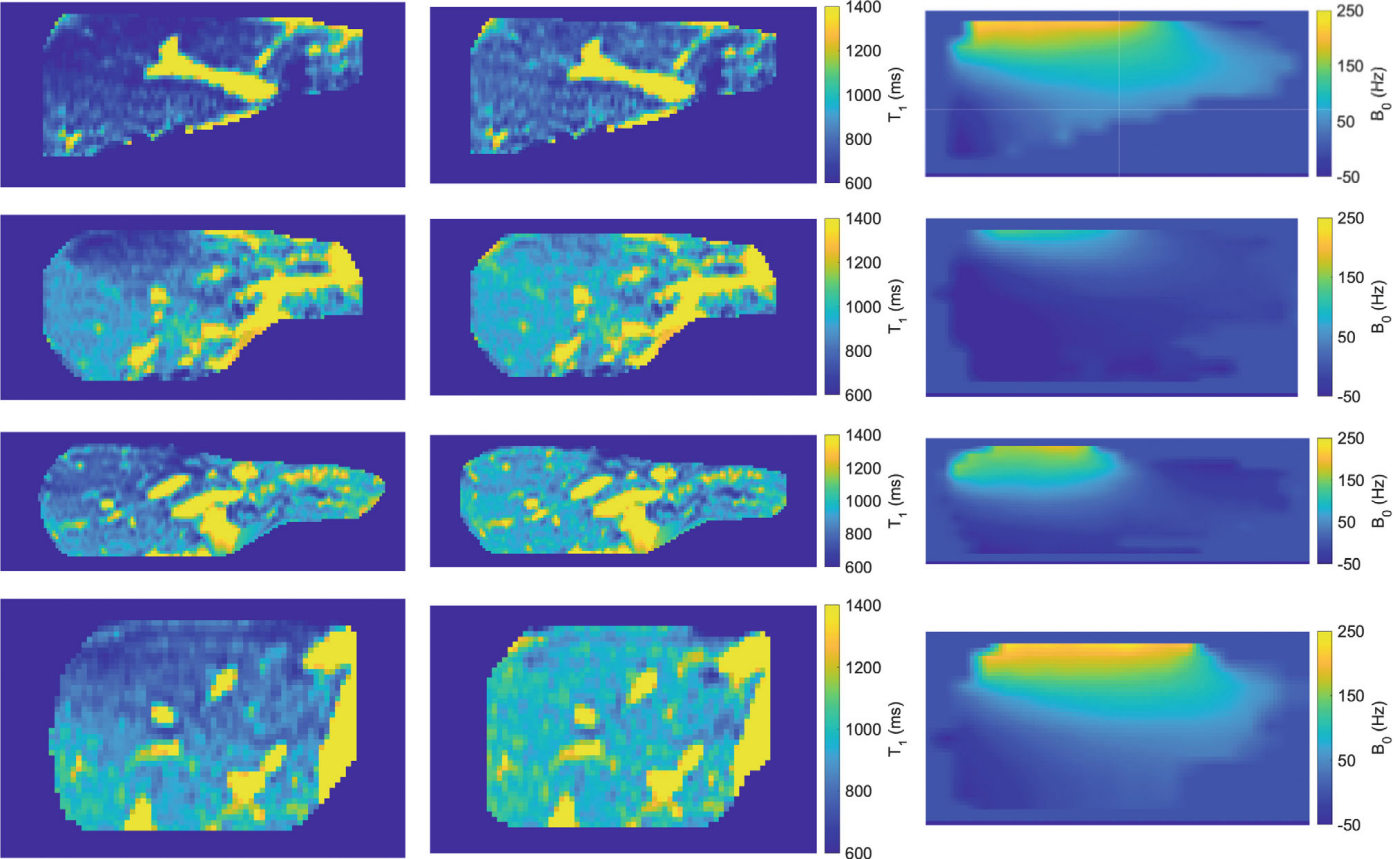
$B_{0}$ gradient through slice effect on in vivo $T_{1}$ maps for four volunteers. Coronal $T_{1}$ maps without the $B_{0}$ gradient through slice correction (first column) show a gradient in $T_{1}$ as a function of slice number (vertical direction), with lower $T_{1}$ values for slices close to the liver dome and increasing $T_{1}$ values as the slice number increased. Applying the developed $B_{0}$ gradient through slice correction (second column), the $T_{1}$ values at the dome of the liver increased, resulting in a more homogeneous $T_{1}$ map. Coronal $B_{0}$ maps showing a large variation in $B_{0}$ values across the liver (third column). Data for all volunteers is in Figure S7.

Figure 4 shows coronal cuts of the in vivo $T_{1}$ maps before and after the $\nabla_{z}B_{0}$ correction for four volunteers. $T_{1}$ maps for all 10 volunteers are in the Supporting Information (Figure S7). The $T_{1}$ is expected to be homogenous throughout the whole liver for a healthy population. After applying the developed correction, the $T_{1}$ gradient disappeared and the $T_{1}$ maps were visually more homogeneous.

Without a $\nabla_{z}B_{0}$ correction, an average difference in $T_{1}$ of 90 ms was observed between the superior and inferior parts of the liver and reached a maximum of 221 ms. After applying the $\nabla_{z}B_{0}$ correction, the variation in $T_{1}$ through slice reduced to between −44 ms and 26 ms (Table 1). For eight out of 10 subjects, the 95% confidence interval in the $∆T_{1}$ included zero. The linear mixed model gave a *p*‐value <10^−6^ for the regressor coefficient of the gradient in $B_{0}$ ($\beta_{1}$in Equation 4), confirming that the $\nabla_{z}B_{0}$ significantly affects the uncorrected $T_{1}$. After applying the proposed correction, the *p*‐value is 0.14 (>0.05).

**TABLE 1:** Average $T_{1}$ for each subject before and after applying the $B_{0}$ gradient through slice correction to the $B_{1}^{+}$ map, which is subsequently used to correct the SPGR excitation FAs.

Subject	Mean $T_{1}$ before $\nabla_{z}B_{0}$ correction (ms)	Mean $T_{1}$ after $\nabla_{z}B_{0}$ correction (ms)	$\Delta T_{1}$ before $\nabla_{z}B_{0}$ correction (ms (95% CI))	$\Delta T_{1}$ after $\nabla_{z}B_{0}$ correction (ms (95% CI))
1	701	809	94 (73 114)	3 (−26 32)
2	736	824	69 (43 94)	26 (0 51)
3	859	1046	−40 (−81 0)	−44 (−74 –15)
4	851	947	148 (126 171)	−16 (−41 8)
5	835	961	73 (54 91)	22 (3 40)
6	720	783	89 (68 110)	−22 (−47 3)
7	718	813	90 (71 110)	12 (−4 29)
8	855	943	54 (5 103)	−17 (−70 36)
9	739	796	97 (66 128)	2 (−33 38)
10	837	969	221 (202 240)	4 (−20 28)

*Note*: The mean $T_{1}$ increases on average 104 ms over all subjects after applying the correction. Columns 4 and 5 correspond to the variation in $T_{1}$ between the superior and inferior parts of the liver for the 10 volunteers. After the $B_{0}$ gradient through slice correction, the variation of $T_{1}$ with slice number decreased significantly.

Abbreviations: CI, confidence interval; FA, flip angle; SPGR, spoiled gradient recalled echo; $\nabla_{z}B_{0}$, $B_{0}$ gradient through slice.

## 4 DISCUSSION

The $B_{1}^{+}$ DAM approach using a GRE‐EPI sequence was chosen due to its wide availability and ability to provide whole liver coverage within two 10 s breath‐holds. We have shown that the 2D multi‐slice nature of the acquisition and the use of a GRE sequence make it sensitive to slice profile effects and $B_{0}$ variations across the slice direction that affect the $B_{1}^{+}$ factor accuracy. An alternative to the proposed $B_{0}$ postprocessing correction would be to use a SE sequence, although slice profile effects would still be relevant. However, not all manufacturers offer SE‐EPI sequences with control over the FAs. Moreover, GRE‐EPI is available even at 7 T, where 180° refocusing pulses may be inaccessible. Rapid $B_{1}^{+}$ DAM has also been proposed for abdominal and cardiac applications. Nonselective saturation pulses^27^ or a catalyzation RF pulse chain^28^ reset the longitudinal magnetization after each TR and thus permit short TRs without a $T_{1}$ bias.

Phantom studies showed that, using the Hamming‐windowed sinc excitation pulse, a median $T_{1}$ error of 10.5% was incurred when using the DAM without slice profile correction, as often presented in the literature.^5^, ^27^, ^29^, ^30^, ^31^ After applying the developed slice profile and $\nabla_{z}B_{0}$ corrections to the $B_{1}^{+}$ map, the median error relative to the GS dropped to 0.01%. Applying the corrected $B_{1}^{+}$ map to the SPGR FAs resulted in accurate $T_{1}$ values with a median error of −0.3%. The $\nabla_{z}B_{0}$ correction greatly improved the homogeneity of in vivo liver $T_{1}$ maps. The mean $T_{1}$ difference across the liver volume, along the slice direction, for the 10 volunteers decreased from 90 ms to −3 ms.

Malik et al.^15^ corrected slice profile effects in 2D actual FA imaging^32^ $B_{1}^{+}$ maps by Bloch‐simulating the signal for the slice‐selective RF excitation pulse and generating an offline LUT. This approach is identical to that adopted in this work to correct for slice profiles alone. Wang et al.^14^ studied both the effect of the RF pulse shape and off‐resonance on estimating the $B_{1}^{+}$ factor using the GRE DAM. The authors reported differences in the actual FA measured across a phantom for three different RF pulse profiles and attributed these to slice profile effects. The authors calculated the average FA across the slice using the DAM formula without slice profile correction (Equation 1) and compared it to the nominal FA to calibrate for slice profiles. This scheme assumes that the average $B_{1}^{+}$ factor in a slice is 1, which is not guaranteed either in vivo or in a phantom. The authors only studied $B_{0}$ offsets and found variations of the FA map of less than 4% using an 800 Hz off‐resonance RF pulse, and of less than 1% for $B_{0}$ inhomogeneities of ±50 Hz. Thus, the authors concluded, as do we, that off‐resonance effects have no impact on the $B_{1}^{+}$ map.

More recently, Nöth et al.^33^ also showed that $B_{0}$ distortions affect the accuracy of the 2D‐DAM GRE‐EPI. The $B_{1}^{+}$ correction consisted of a seventh‐order 2D polynomial that depended on the time BW product of the RF pulse and the $B_{0}$ gradient value. Their correction method does not account for the effect of the $B_{0}$ gradient during RF excitation. As shown by our Bloch simulations (Figure 2), the differences in dephasing between the α and 2α during excitation are crucial to explain the effect of $B_{0}$ variations through slice on the DAM ratio and consequently the error in the $B_{1}^{+}$ map. The dephasing will proceed during the slice refocusing gradient until TE but should already be considered during excitation.

Several limitations are present in the Nöth et al.^33^ method. The polynomial coefficients were calculated based on a FA pair of 45°/90°. As shown in our previous work, this is not the optimal FA pair for DAM GRE‐EPI acquisition.^18^ Presumably, the polynomial coefficients need to be recalculated when using different FA pair values, whereas our algorithm is valid for any combination of FAs. Moreover, the authors mention their method is not valid when the RF pulse shape is angle‐dependent. This is not the case for our algorithm. For example, our algorithm would still work if the pulse duration is increased instead of scaling the pulse amplitude. Related to this, Nöth et al.'s^33^ correction is independent of the $B_{1}^{+}$ factor, whereas we show in Figure 2C that this is not the case, at least when the two FAs have different slice profiles. Nöth et al. results show some $B_{1}^{+}$ overestimations, and a residual $B_{1}^{+}$ gradient through slice seems to persist after the $B_{0}$ correction. This may be due to the use of a constant $B_{0}$ gradient for each slice position. We found it is important to take into account higher order variations and interpolate the $B_{0}$ values within a slice because this will improve the accuracy of the $B_{1}^{+}$ maps, and ultimately the VFA $T_{1}$ maps. This effect should become more important as the slice thickness increases.

The biasing effect of $\nabla_{z}B_{0}$ on $B_{1}^{+}$ mapping will affect any 2D multislice GRE DAM, including the saturated DAM.^26^ In addition, slice profile effects and $B_{1}^{+}$ inhomogeneities are important in MR fingerprinting.^6^ The correction reported here may also improve the accuracy of MR fingerprinting measurements given that they primarily use 2D GRE acquisitions.

Our method requires an accurate knowledge of the $B_{0}$ field along the slice direction. When there is no information on $B_{0}$ values in slices above the target pixel where the $B_{1}^{+}$ factor is being calculated, the $B_{0}$ extrapolation may not be accurate. Investing in a higher resolution $B_{0}$ map sequence extending beyond the $B_{1}^{+}$ map FOV coverage along the slice direction could help. However, areas with low signal and susceptible to respiratory motion will always be prone to inaccurate and imprecise $B_{0}$, and thus $B_{1}^{+}$ map values. Another limitation toward adopting the proposed correction is the added computational time, which increases with the acquisition matrix size.

## 5 CONCLUSIONS

Through‐slice gradients in $B_{0}$ are a key confounder affecting the accuracy of GRE 2D dual‐angle $B_{1}^{+}$ maps. Novel postprocessing corrections to the GRE data for this confounder resulted in accurate $B_{1}^{+}$ and SPGR $T_{1}$ maps in phantom data. In vivo correction for this confounder is crucial in areas susceptible to large $B_{0}$ inhomogeneities. Failure to consider the impact of $\nabla_{z}B_{0}$ on the $B_{1}^{+}$ map acquisition resulted in $T_{1}$ variations between the top and bottom of the liver of up to 221 ms. After correcting for $\nabla_{z}B_{0}$, the average $T_{1}$ variation decreased to −3 ms.

## Supporting information


**Figure S1.** Ratio between the signals at 2α and α for an ideal rectangular slice profile (green) and a non‐uniform slice profile using a Hamming Windowed Sinc (HWS) excitation pulse (blue). The ratio is interpolated (blue dashed arrows) to find the true FA exciting the spins and calculate the $B_{1}^{+}$ correction factor. Without slice profile effects the ambiguity angle is equal to 90°, with the HWS slice profile the ambiguity angle increases to 95°.
**Figure S2.** Accuracy of the 3D $T_{1}$ SPGR method on the phantom. SPGR $T_{1}$ using the slice profile and the $B_{0}$ gradient through slice corrections developed as a function of the $T_{1}$ gold standard inversion recovery spin echo in the phantom for 14 different vials across 4 slices. Linear fit, in red, resulted in a slope of 1.026 ms with an intercept of −27.76 ms. The $R^{2}$ was 0.996.
**Figure S3.** Inversion‐recovery spin echo gold standard $T_{1}$ map of the phantom with mean $T_{1}$s varying between 367 ms and 1699 ms.
**Figure S4.** $B_{1}^{+}$ gold standard maps of the phantom across 16 slices.
**Figure S5.** Validation of the gold standard $B_{1}^{+}$ mapping method. (A) SPGR $T_{1}$ map with the SPGR FAs corrected by the 3D GRE $B_{1}^{+}$ map. The signal was corrected for incomplete spoiling. (B) Validation of the gold standard $B_{1}^{+}$ mapping method. Linear fit of the SPGR $T_{1}$ map to the gold standard IR SE. The slope from the linear fit was 1.03 with a 95% confidence interval between 1.019 and 1.046.
**Figure S6.** Phantom $B_{1}^{+}$ map (A) without and (B) with the $B_{0}$ gradient through slice correction. (C) Difference between the corrected and uncorrected $B_{1}^{+}$ maps. Note the presence of the vials in the $B_{1}^{+}$ map without the correction, highlighting a bias as the $B_{1}^{+}$ should vary smoothly across space and present no structure. Differences in $B_{1}^{+}$ close to 10% were seen in the centre of most vials which results in $T_{1}$ differences of 20%. Corresponding phantom $T_{1}$ maps using the $B_{1}^{+}$ maps (D) without and (E) with the $B_{0}$ gradient through slice correction. (F) Difference between the corrected and uncorrected $T_{1}$ maps. Mean absolute differences of more than 100 ms were observed for 10 out of 14 vials and the largest mean absolute difference was 265 ms. The $B_{1}^{+}$ map in (B) or the $T_{1}$ map in (E) do not have a circular border due to the mask used to calculate the $B_{0}$ map and correct distortion in the GRE‐EPI images used in the $B_{1}^{+}$ calculation. $B_{0}$ maps clearly show the vial locations as areas of increased $B_{0}$ compared to the flood region. $B_{0}$ map corresponding to the (H) slice for which the $B_{1}^{+}$/$T_{1}$ maps were calculated, and adjacent slices: (G) superior and (I) inferior to slice (H). Note that the $B_{0}$ is changing significantly through slice and thus the necessity of applying the correction we developed.
**Figure S7.** Coronal $T_{1}$ maps without the $B_{0}$ gradient through slice correction show a gradient in $T_{1}$ through slice (first column). The slice direction is the vertical direction. The $T_{1}$ gradient though slice is reduced after correcting for variations in the $B_{0}$ gradient though slice (second column). Coronal $B_{0}$ maps showing a large variation in the $B_{0}$ inhomogeneity across the liver (third column).
**Figure S8.** $B_{0}$ values extracted from 3 ROIs containing 46 pixels each, drawn across the liver extent in the slice direction for each of the 10 volunteers. Note that the variation in the $B_{0}$ as a function of slice number is steeper for the first slices and becomes almost constant for the end slices. The ROIs were chosen in vessel free areas of the liver and thus were not in the same pixel coordinates for each slice.

## Data Availability

The code for the B_0_ gradient through slice correction and the slice profile effects is available here: https://github.com/gabrielaBelsley/ThroughSliceDephasing_2DGRE.
