# Supplementary material for: The effect of and correction for through‐slice dephasing on 2D gradient‐echo double angle B1+ mapping
Source: Magn Reson Med. 2023 Dec 29;91(4):1598–607. doi: 10.1002/mrm.29966 (PMC10952755; doi:10.1002/mrm.29966)
Supplement: Supplementary file 1 — Figure S1. Ratio between the signals at 2α and α for an ideal rectangular slice profile (green) and a non‐uniform slice profile using a Hamming Windowed Sinc (HWS) excitation pulse (blue). The ratio is interpolated (blue dashed arrows) to find the true FA exciting the spins and calculate the B1+ correction factor. Without slice profile effects the ambiguity angle is equal to 90°, with the HWS slice profile the ambiguity angle increases to 95°. Figure S2. Accuracy of the 3D T1 SPGR method on the phantom. SPGR T1 using the slice profile and the B0 gradient through slice corrections developed as a function of the T1 gold standard inversion recovery spin echo in the phantom for 14 different vials across 4 slices. Linear fit, in red, resulted in a slope of 1.026 ms with an intercept of −27.76 ms. The R2 was 0.996. Figure S3. Inversion‐recovery spin echo gold standard T1 map of the phantom with mean T1s varying between 367 ms and 1699 ms. Figure S4. B1+ gold standard maps of the phantom across 16 slices. Figure S5. Validation of the gold standard B1+ mapping method. (A) SPGR T1 map with the SPGR FAs corrected by the 3D GRE B1+ map. The signal was corrected for incomplete spoiling. (B) Validation of the gold standard B1+ mapping method. Linear fit of the SPGR T1 map to the gold standard IR SE. The slope from the linear fit was 1.03 with a 95% confidence interval between 1.019 and 1.046. Figure S6. Phantom B1+ map (A) without and (B) with the B0 gradient through slice correction. (C) Difference between the corrected and uncorrected B1+ maps. Note the presence of the vials in the B1+ map without the correction, highlighting a bias as the B1+ should vary smoothly across space and present no structure. Differences in B1+ close to 10% were seen in the centre of most vials which results in T1 differences of 20%. Corresponding phantom T1 maps using the B1+ maps (D) without and (E) with the B0 gradient through slice correction. (F) Difference between the corrected and uncorrected [file MRM-91-1598-s001.docx]

**Supporting Information**

$\boldsymbol{B}_{\boldsymbol{1}}^{\boldsymbol{+}}$ **Factor Look-Up-Table**

 Figure S1 illustrates the look-up-table used to calculate the $B_{1}^{+}$ factor corrected for slice profile effects from the ratio of two fully relaxed GRE signals acquired at FAs $2\alpha$ and $\alpha$.

Figure S1. ﻿Ratio between the signals at 2$\alpha$ and $\alpha$ for an ideal rectangular slice profile (green) and a non-uniform slice profile using a Hamming Windowed Sinc (HWS) excitation pulse (blue). The ratio is interpolated (blue dashed arrows) to find the true FA exciting the spins and calculate the $B_{1}^{+}$ correction factor. Without slice profile effects the ambiguity angle is equal to 90°, with the HWS slice profile the ambiguity angle increases to 95°.

$\boldsymbol{T}_{\boldsymbol{1}}$ **Map Validation using a Gold Standard** $\boldsymbol{T}_{\boldsymbol{1}}$ **Map**

A gold standard (GS) $T_{1}$ map using a slice-selective inversion-recovery spin echo (IR SE) was acquired on the phantom using TIs of [25, 50, 75, 100, 200, 300, 400, 600, 800, 1200, 1600, 2300, 3000, 4000, 5000]ms, TR/TE=9s/12ms, FoV=$225\times300 mm^{2}$, matrix=$144\times192$, 6mm slice thickness, BW=130 Hz/pixel, Phase Partial Fourier Off, Interpolation On, no acceleration. The acquisition time for each TI was 21 minutes and 47 seconds.

Applying the slice profile and the $B_{0}$ gradient through slice (${\nabla_{z}B}_{0}$) corrections developed to the $B_{1}^{+}$ map, subsequently used to correct the SPGR FAs, resulted in a final $T_{1}$ map in the phantom showing an excellent agreement with the GS IR SE (Figure S2). The weighted least squares linear fit between the SPGR $T_{1}$, for all 14 vials across 4 slices, and the GS IR SE resulted in a slope of 1.026 with a 95% CI between 1.008 and 1.043. Across the physiologically relevant liver $T_{1}$ values at 3T (740-1211ms), the IQR relative $T_{1}$ error was [-2.3 0.4]%. The weighted root mean squared normalised error between the SPGR $T_{1}$ and the GS $T_{1}$ was 2.5%. The weights were equal to the inverse of the SPGR $T_{1}$ standard deviation squared.

Figure S2. Accuracy of the 3D $T_{1}$ SPGR method on the phantom. SPGR $T_{1}$ using the slice profile and the $B_{0}$ gradient through slice corrections developed as a function of the $T_{1}$ gold standard inversion recovery spin echo in the phantom for 14 different vials across 4 slices. Linear fit, in red, resulted in a slope of 1.026 ms with an intercept of -27.76 ms. The $R^{2}$ was 0.996.

To calculate the GS $T_{1}$ map (Figure S3), the signal in each pixel was fit using a three-parameter non-linear IR model:

$$S_{n}=a-be^{\left( \frac{-TI_{n}}{T_{1}} \right)} ,$$

where $S_{n}$ is the signal intensity, $a$ is the signal at $TI=\infty$, after its full recovery to the equilibrium longitudinal magnetisation $M_{z}$, and $a-b$ is the signal at $TI=0$. Initial guesses for $a$ is the signal from the largest TI (5000 ms) and for $b$ is two times the signal at the largest TI. $T_{1}$ was initialised to 500 ms. The subscript $n$ denotes the index of the TI; in this work the signal was measured at 15 different TIs. For each fit, the TI corresponding to the minimum signal intensity is determined and all the TIs less than or equal to the TI with the minimum signal intensity are assigned a negative value, i.e., samples which have not reached the null intensity. A second fit is carried out with the minimum signal intensity changed back to positive. The fit with the smallest sum squared errors value is chosen.

Figure S3. Inversion-recovery spin echo gold standard $T_{1}$ map of the phantom with mean $T_{1}$s varying between 367 ms and 1699 ms.

$\boldsymbol{B}_{\boldsymbol{1}}^{\boldsymbol{+}}$ **Map Validation using a Gold Standard** $\boldsymbol{B}_{\boldsymbol{1}}^{\boldsymbol{+}}$ **Map**

A 3D non-selective GRE sequence gave a GS $B_{1}^{+}$ map using the DAM. Two acquisitions at nominal FAs of 30˚ and 60˚ were acquired in an interleaved manner to avoid signal drift over time. Acquisition parameters were: FoV=$192\times192\times10 mm^{3}$, matrix=$48\times48\times16$, TR/TE=10s/2ms, BW=1000 Hz/pixel, Slice/Phase Partial Fourier Off, Interpolation On, no acceleration. The acquisition time for each FA was 4 hours and 16 minutes.

Figure S4 shows the $B_{1}^{+}$ GS maps in the phantom. Given the central slices in 3D acquisitions have a rectangular or ideal slice profile, Eq. 1 in the paper was used to calculate the true FAs exciting the spins in the phantom.


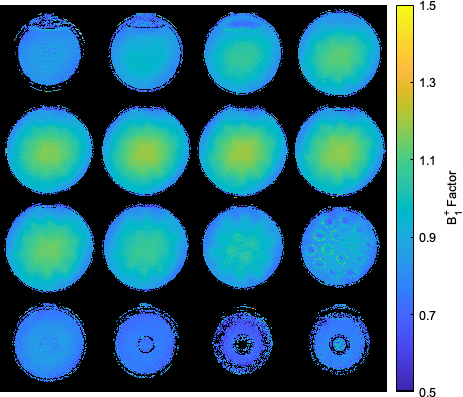


Figure S4. $B_{1}^{+}$ gold standard maps of the phantom across 16 slices.

To check whether the 3D GRE DAM $B_{1}^{+}$ could be used as the gold standard $B_{1}^{+}$ mapping method in the phantom, the SPGR $T_{1}$ map with the FAs corrected by the 3D GRE DAM $B_{1}^{+}$ was compared to the IR SE $T_{1}$ map. A good agreement was achieved with the IR SE, as shown in Figure S5 with a slope close to the identity line.

Figure S5. Validation of the gold standard $B_{1}^{+}$ mapping method. (a) SPGR $T_{1}$ map with the SPGR FAs corrected by the 3D GRE $B_{1}^{+}$ map. The signal was corrected for incomplete spoiling. (b) Validation of the gold standard $B_{1}^{+}$ mapping method. Linear fit of the SPGR $T_{1}$ map to the gold standard IR SE. The slope from the linear fit was 1.03 with a 95% confidence interval between 1.019 and 1.046.

The temperature of the 3D SPGR $T_{1}$ mapping protocol and the GS $B_{1}^{+}$ and $T_{1}$ mapping was monitored overnight using fibre optic probes attached to the surface of the phantom. The mean ± standard deviation temperature was 20±0.4$℃$ over the duration of 19 hours. The magnitude of these temperature variations result in insignificant $T_{1}$ changes for NiCl_2_ filled vials^1^. Stupic et al.^1^ measured $T_{1}$ variations of 1.3% in a NiCl_2_ array over a temperature range from 18$℃$ to 26$℃$.

**Phantom 3D SPGR** $\boldsymbol{T}_{\boldsymbol{1}}$ **Maps**

Figure S6 shows the phantom $B_{1}^{+}$ maps and the corresponding $T_{1}$ maps before and after ${\nabla_{z}B}_{0}$ correction for SPGR slice 32. The phantom contained 14 vials with $T_{1}$s (at 3 T) ranging from 367 ms to 1699 ms in steps of approximately 100 ms, an average T2 of 45 ms and no fat. Note the structure present in the $B_{1}^{+}$ map in (a), suggesting an underlying bias as the $B_{1}^{+}$ map is expected to vary smoothly as seen in (b) after the ${\nabla_{z}B}_{0}$ correction. The $B_{1}^{+}$ difference map shows that the $B_{1}^{+}$ factor after the correction was larger in the vials, i.e. using only slice profile correction there is an underestimation of $B_{1}^{+}$ factor which translates into a $T_{1}$ overestimation. In conclusion, correcting for slice profile effects is not enough to obtain accurate $B_{1}^{+}$ and $T_{1}$ maps.


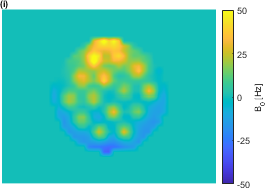

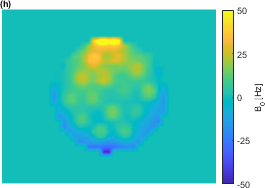

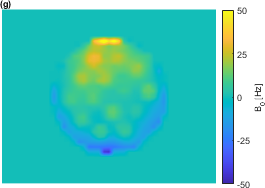


Figure S6. Phantom $B_{1}^{+}$ map (a) without and (b) with the $B_{0}$ gradient through slice correction. (c) Difference between the corrected and uncorrected $B_{1}^{+}$ maps. Note the presence of the vials in the $B_{1}^{+}$ map without the correction, highlighting a bias as the $B_{1}^{+}$ should vary smoothly across space and present no structure. Differences in $B_{1}^{+}$ close to 10% were seen in the centre of most vials which results in $T_{1}$ differences of 20%. Corresponding phantom $T_{1}$ maps using the $B_{1}^{+}$ maps (d) without and (e) with the $B_{0}$ gradient through slice correction. (f) Difference between the corrected and uncorrected $T_{1}$ maps. Mean absolute differences of more than 100 ms were observed for 10 out of 14 vials and the largest mean absolute difference was 265 ms. The $B_{1}^{+}$ map in (b) or the $T_{1}$ map in (e) don’t have a circular border due to the mask used to calculate the $B_{0}$ map and correct distortion in the GRE-EPI images used in the $B_{1}^{+}$ calculation. $B_{0}$ maps clearly show the vial locations as areas of increased $B_{0}$ compared to the flood region. $B_{0}$ map corresponding to the (h) slice for which the $B_{1}^{+}$/$T_{1}$ maps were calculated, and adjacent slices: (g) superior and (i) inferior to slice (h). Note that the $B_{0}$ is changing significantly through slice and thus the necessity of applying the correction we developed.

***In Vivo* 3D SPGR** $\boldsymbol{T}_{\boldsymbol{1}}$ **Maps**

Figure S7 shows coronal $T_{1}$ maps before and after applying the ${\nabla_{z}B}_{0}$ correction for all volunteers, together with the $B_{0}$ map.

Figure S7. Coronal $T_{1}$ maps without the $B_{0}$ gradient through slice correction show a gradient in $T_{1}$ through slice (first column). The slice direction is the vertical direction. The $T_{1}$ gradient though slice is reduced after correcting for variations in the $B_{0}$ gradient though slice (second column). Coronal $B_{0}$ maps showing a large variation in the $B_{0}$ inhomogeneity across the liver (third column).

The liver is a good organ to test the proposed correction as the $B_{0}$ changes significantly in magnitude from the liver dome, next to the diaphragm, towards the posterior part of the liver, next to the kidneys. On average, the $B_{0}$ changed between 130 Hz and 30 Hz across the 10 volunteers along the slice direction (Figure S8). The $T_{1}$ gradient through slice is a consequence of a variation in the ${\nabla_{z}B}_{0}$, i.e. the second derivative of the $B_{0}$ in the slice direction is different from zero. A constant ${\nabla_{z}B}_{0}$ would result in a constant overestimation or underestimation of $T_{1}$. However, the observed $T_{1}$ gradient through slice is a consequence of a change in the $B_{0}$ gradient as can be seen from Figure S8. The first slices, closer to the lungs, have a steeper slope while the end slices have a shallower $B_{0}$ gradient as a function of z.

Figure S8. $B_{0}$ values extracted from 3 ROIs containing 46 pixels each, drawn across the liver extent in the slice direction for each of the 10 volunteers. Note that the variation in the $B_{0}$ as a function of slice number is steeper for the first slices and becomes almost constant for the end slices. The ROIs were chosen in vessel free areas of the liver and thus were not in the same pixel coordinates for each slice.

References

1. Stupic KF, Ainslie M, Boss MA, et al. A standard system phantom for magnetic resonance imaging. *Magn Reson Med*. 2021;86(3):1194-1211. doi:10.1002/mrm.28779
